# Distribution of Summer Zooplankton in the Waters off the Kuril Islands (Northwest Pacific) in Relationship with Environmental Conditions

**DOI:** 10.3390/biology14070827

**Published:** 2025-07-08

**Authors:** Valentina Kasyan

**Affiliations:** Laboratory of Systematics and Morphology, A.V. Zhirmunsky National Scientific Center of Marine Biology, Far Eastern Branch, Russian Academy of Sciences (NSCMB FEB RAS), Vladivostok 690041, Russia; valentina-k@yandex.ru

**Keywords:** zooplankton, composition, abundance, biomass, distribution, environmental factors, Kuril Islands

## Abstract

This study covers a large area around the Kuril Islands, one of the longest island arcs in the world, crossing several climatic zones, which allows for observations of longitudinal shifts in planktonic species’ ranges following shifts in the boundaries between the climatic zones. We analyzed spatial and vertical changes in the zooplankton community structure and the associated environmental factors from Yuri Island to Onekotan Island both in Pacific and Sea of Okhotsk waters, which are influenced by cold and warm water masses. Species diversity in the Pacific waters was higher than in the Sea of Okhotsk waters, with a peak of diversity recorded from warm waters off the southern Kuril Islands associated with the Soya Current and the Kuroshio Extension. Zooplankton abundance and biomass above the thermocline were higher in the Pacific waters compared to the Sea of Okhotsk area, showing a tendency to increase with higher latitude and lower water temperatures and generally to decline with depth. The water temperature and salinity below the thermocline were the most important explanatory environmental variables responsible for zooplankton abundance variations. The distribution patterns of the large-sized copepods were strongly correlated with temperature and salinity, as well as with dissolved oxygen and chlorophyll *a* levels. In contrast, small-sized copepods were adapted to or thrived in the areas with elevated temperature and salinity values and a reduced chlorophyll *a* concentration.

## Simple Summary

In this study, we investigated the key environmental factors driving longitudinal changes in zooplankton assemblages around the Kuril Islands, Northwest Pacific. The spatial and vertical variations in the composition of zooplankton assemblages were attributed to parameters of water masses. The water temperature and salinity below the thermocline were the primary drivers of zooplankton abundance. The same environmental factor might have different effects on the abundance of boreal species. These results enhance our understanding of how marine environment conditions cause changes in planktonic communities and highlight the ecological importance of zooplankton as a natural indicator of environmental quality.

## 1. Introduction

The Kuril Islands are part of the Pacific Ring of Fire which consists of the largest number of active volcanoes on the planet. Millions of years ago, eruptions of underwater volcanoes in the Pacific Ocean formed large and small patches of land known today as the Kuril Islands. These have become a kind of barrier separating the Sea of Okhotsk from the Pacific Ocean and one of the longest island arcs in the world stretching in the longitudinal direction and crossing several climatic zones, from temperate to subarctic. The area around the islands is home to numerous endemic animal species. The waters off the Kuril Islands, being part of the northwestern Pacific Ocean, are a highly productive region that provides habitats and feeding grounds for commercially valuable boreal, subtropical, and tropical planktivorous fishes, cephalopods, and migrating Pacific salmon [1,2,3,4]. Their food supply depends largely on the abundance of zooplankton. The most important zooplankton prey species include the euphausiids *Thysanoessa longipes*, *Thysanoessa inermis*, and *Euphausia pacifica*, the hyperiid *Themisto pacifica*, the copepods *Neocalanus cristatus* and *Neocalanus plumchrus*, the pteropods *Clione limacina* and *Limacina helicina*, and the chaetognaths *Parasagitta elegans*. Any changes in zooplankton abundance may affect the diet and food supply for nekton [5]. Additionally, the waters along the Kuril Islands, referred to as “copepods waters” [6], are an important nursery ground for animals of higher trophic levels such as whales and seabirds [7,8,9].

The waters off the Kuril Islands are characterized by intensive dynamics and a variety of oceanographic conditions [10,11,12], resulting in significant spatial variations in both qualitative and quantitative characteristics of zooplankton. The sea currents along the Kuril Islands, including the cold Oyashio Current (OC), flowing south, and the warm Soya Current (SC), flowing north, have a pronounced effect on the regional circulation of water masses [13,14,15,16]. In recent decades, thermal conditions in the waters along the Kuril Islands have changed [10,17,18] due to global atmospheric processes [11,19]. Changes in the hydrological regime are typically accompanied by shifts in the marine ecosystem, thereby influencing the structure of zooplankton communities [20,21,22,23,24]. For example, in recent decades, the latitudinal gradient of species diversity in the Sea of Okhotsk was disrupted [25,26]. The 30-year dynamics of the pelagic communities in the southern Kuril Islands showed a downward trend in the biomass of copepods and euphausiids [27,28]. The exceptions were the copepods *Oithona* and amphipods whose abundance remained stable [29].

The pelagic communities in the Kuril Islands region have been described in detail through both single-season studies and the long-term monitoring of fish stocks [9,27,30,31,32,33], while data on the ecological relationships between environmental characteristics and the quantitative distribution of zooplankton are still scarce and fragmentary [30,34]. Most of these studies were based on total sampling within the epipelagic layer (0–200 m). However, we here propose a method with division of vertical sampling into zones above and below the thermocline because the zooplankton communities may vary significantly with depth, especially in the area of the southern Kuril Islands where cold and warm surface currents mix. Additionally, our study underscores the need to identify species among boreal copepods that could serve as environmental indicators. The aim of our study was to clarify the relationships between the quantitative distribution of zooplankton and the environmental conditions along the Kuril Islands. We hypothesized that the shifts in water mass distribution, thermal structure, and current dynamics along the Kuril Islands markedly affected the distribution of zooplanktonic organisms in terms of spatial extent and relative abundance/biomass, which changed the dominance of boreal species. We investigated the zooplankton species composition and the spatial and vertical distribution patterns of abundance and biomass along the Kuril Islands, from Yuri Island (42° N) to Onekotan Island (49° N), in both the Pacific and Sea of Okhotsk waters using plankton samples collected from two vertical zones: above and below the thermocline. Although this study is based on a single survey and, thus, represents changes within one seasonal cycle, it helps establish a baseline for zooplankton monitoring and research in Kuril Islands ecosystems. The more we know about zooplankton, the more effectively we can manage marine biological resources and take appropriate measures for their conservation and sustainable use.

## 2. Materials and Methods

### 2.1. Field Observations

**Figure 1:**
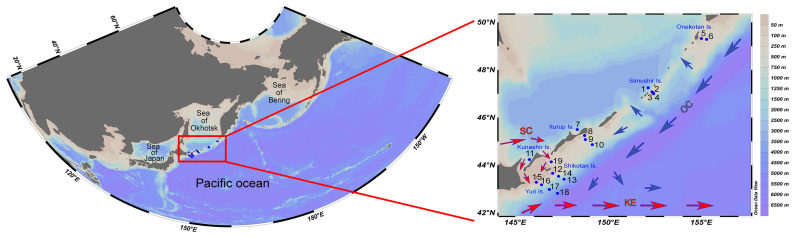
Map of sampling localities and surface currents along the Kuril Islands, including Soya Current (SC); Kuroshio Extension (KE); and Oyashio Current (OC). Blue dots with numerals indicate zooplankton sampling stations. Approximate flow directions of the surface currents are indicated by arrows. Blue arrows indicate cold currents; red arrows, warm currents.

Sampling was conducted at 19 stations onboard the R/V Akademik Oparin from 17 August to 10 September 2024, along the Kuril Islands from Yuri Island (southern Kuril Islands) to Onekotan Island (northern Kuril Islands) in both Pacific (stns. 2–6, 8–10, and 12–18) and Sea of Okhotsk (stns. 1, 7, 11 and 19) waters (Figure 1). Zooplankton was collected using a WP2 net (0.25 m^2^ mouth opening, 168 μm mesh size), towed vertically in two depth zones: above the thermocline (∼0–50 m) and below the thermocline (∼50–200 m). In areas shallower than 100 m, the entire water column from the bottom to the surface was sampled. After towing, the samples were fixed in a 4% formaldehyde solution in seawater. Environmental parameters such as water temperature (T, °C), salinity (S, psu), density (D, kg m^−3^), dissolved oxygen concentration (O, mL L^−1^), and chlorophyll *a* concentration (Chla, µg L^−1^) for the water column were recorded for 1 m intervals and binned using a CTD profiler (SBE 19plus V2, Sea-Bird Scientific Co., Washington, DC, USA) at each station. Chlorophyll *a* fluorescence intensity was measured using a Seapoint Chlorophyll Fluorometer mounted on the CTD profiler. The depth of the thermocline was estimated for each station by locating the depth where the temperature difference between the upper and lower boundaries was more than 5 °C. The mean values of the temperature (T_up_), salinity (S_up_), density (D_up_), dissolved oxygen concentration (O_up_), and chlorophyll *a* concentration (Chla_up_) above the thermocline and values of the same parameters (T_lo_, S_lo_, D_lo_, O_lo_, and Chla_lo_) below the thermocline were then computed.

### 2.2. Sample Processing

In the laboratory, the samples were examined under an SZX7 stereo microscope (Olympus Co., Tokyo, Japan), and all individuals were identified to the lowest taxonomic level possible. Species synonymy was according to the World Register of Marine Species [35]. Copepods, as the dominant zooplankton group, were subjected to a more detailed taxonomic analysis using the Marine Planktonic Copepods database [36]. Copepods were identified to species or genus level (*Oithona similis*, *O. plumifera*, and *Oithona* sp. were grouped as *Oithona* spp.); most other groups were classified as broad taxonomic categories. Copepodite stages C1–C6 (adult) were identified for the boreal species *Neocalanus cristatus*, *Neocalanus plumchrus*, *Eucalanus bungii*, *Pseudocalanus minutus*, *Pseudocalanus newmani*, and *Metridia pacifica*, while other copepod species were referred to as adults/copepodites. Large individuals (>2000 μm) were counted in the whole sample, while smaller and more abundant taxa were counted in sub-samples of 1/5 to 1/100. The abundance of zooplankton was calculated by dividing the total number of individuals by the filtered water volume and was expressed as individuals per cubic meter (ind. m^−3^). Biomass was calculated by multiplying the number of individuals by the wet weight of each individual according to Borisov et al. [37] and expressed as milligrams per cubic meter (mg m^−3^).

### 2.3. Data Analysis

A non–metric multidimensional scaling (nMDS) analysis [38] was used to identify and illustrate the multivariate zooplankton community structure in the study region. Ordination was carried out using the Bray–Curtis dissimilarities of square-root transformed species abundances. Cluster analysis was applied in the mode of average group clustering. The SIMPER (Similarity Percentage) procedure was used to estimate the average percentage contribution of each taxon to the overall dissimilarity, the contribution of each taxon to the average intra-group similarity (Sim) with standard deviation (SD) (Sim/SD ratio > 3), and the contribution of each taxon to the average between-group dissimilarity (Diss/SD). The species diversity was calculated using the Shannon–Wiener index (H′), and the species richness using Margalef’s index (D′); for estimating the evenness of species, Pielou’s evenness index (J′) was used [39].

Relationships between the biomass of major groups of zooplankton taxa and the environmental parameters above and below the thermocline at each station were assessed using the Spearman’s rank order correlation. The BEST analysis (BIOENV algorithm) was used to identify the tested environmental variables (water temperature, salinity, density, oxygen, and chlorophyll *a*) that best explain the observed patterns in zooplankton distribution [40]. The environmental parameters were logarithmically transformed, normalized (divided by standard deviation), and expressed as Euclidean distances. The RELATE test was applied to determine the significance of any relationship between the similarity matrices underlying the biological data and the environmental parameters. Additionally, Canonical Correspondence Analysis (CCA) was conducted to determine the correlation between boreal copepods, which accounted for more than 50% of the total biomass, and environmental conditions. The multivariate analyses were carried out using PRIMER [41] and PAST [42]. Maps and species distributions were plotted using the Ocean Data View [43].

## 3. Results

### 3.1. Environments

**Figure 2:**
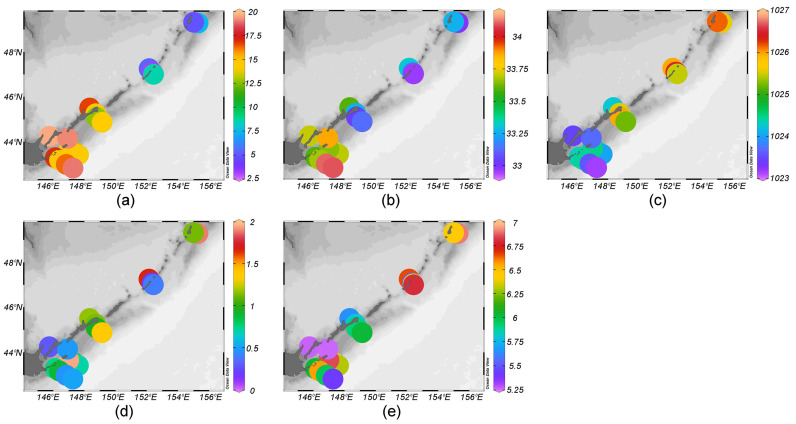
Water (**a**) temperature, °C; (**b**) salinity, psu; (**c**) density, kg m^−3^; (**d**) chlorophyll *a* concentration, µg L^−1^; and (**e**) dissolved oxygen concentration, mL L^−1^ above the thermocline along the Kuril Islands.

The oceanographic conditions around the Kuril Islands were characterized by significant heterogeneity because of intensive water dynamics, the intrusion of warm subtropical waters via the Soya Current (SC) and the Kuroshio Extension (KE) into the southern parts, and the intrusion of cold boreal waters via the Oyashio Current (OC) into the northern parts of the study region [13]. Water temperature, salinity, and density varied depending on station (stn.) and associated currents. In the zone of OC influence (stns. 1–6), the water temperature above the thermocline increased from Onekotan Island to Simushir Island (2.7–6.6 °C) (Figure 2a). Below the thermocline, the OC flowed southward from Onekotan Island to Yuri Island with low temperature (1.3–5.9 °C) and salinity values (32.9–33.5 psu). Relatively high salinity above the thermocline (33.6–33.9 psu) was recorded from both the Pacific (stns. 12, 14–16) and Sea of Okhotsk (stns. 7, 11 and 19) areas off the southern Kuril Islands, with values characteristic of the warm SC [15]. The highest temperature and salinity above the thermocline were recorded from the waters off Yuri Island, influenced by the KE [13], which formed well-stratified transition zones characterized by denser, cold (<5 °C), and saline (~33 psu) water in the near-bottom layer. The temperature above the thermocline ranged from 2.7 to 19.5 °C (Figure 2a), the salinity between 32.9 and 34.1 psu (Figure 2b), the density from 1023 to 1027 kg m^−3^ (Figure 2c), the chlorophyll *a* concentration from 0.3 to 2.6 µg L^−1^ (Figure 2d), and the dissolved oxygen concentration between 5.1 and 7.3 mL L^−1^ (Figure 2e). The warmest oceanographic conditions were observed off the southern Kuril Islands, while the coldest conditions were off the northern Kuril Islands.

**Figure 3:**
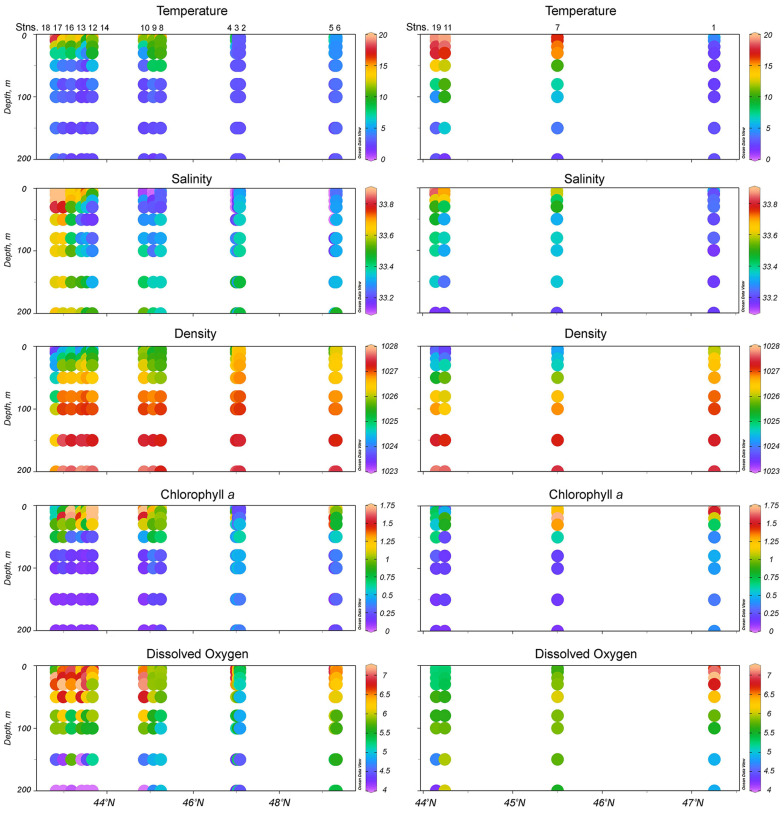
Vertical profiles of temperature, °C; salinity, psu; density, kg m^−3^; chlorophyll *a* concentration, µg L^−1^; and dissolved oxygen concentration, mL L^−1^ at the stations (stns.) in the Pacific (**left column**) and Sea of Okhotsk (**right column**) areas along the Kuril Islands.

The water temperature was the highest off the southern Kuril Islands, where it ranged from 10 to 19 °C in the upper 20–50 m layer (Figure 3). Beneath this maximum, the temperature decreased and remained stable at the level of <6 °C to the bottom. Around the northern Kuril Islands, the temperature below the thermocline was markedly colder and reached 1 °C. A summer thermocline was formed at approximately 20–40 m depth off the northern Kuril Islands, while off the southern Kuril Islands, it was formed at 20–100 m depth. The highest chlorophyll *a* concentrations, suggesting a decrease in SC velocity, were recorded from the Pacific waters off Shikotan Island. Relatively high oxygen concentrations, suggesting local mixing, were observed in the Pacific waters off Shikotan Island, Iturup Island, and Yuri Island and in the Sea of Okhotsk waters off Simushir Island (Figure 3).

### 3.2. Species Composition and Distribution Patterns of Abundance and Biomass

**Figure 4:**
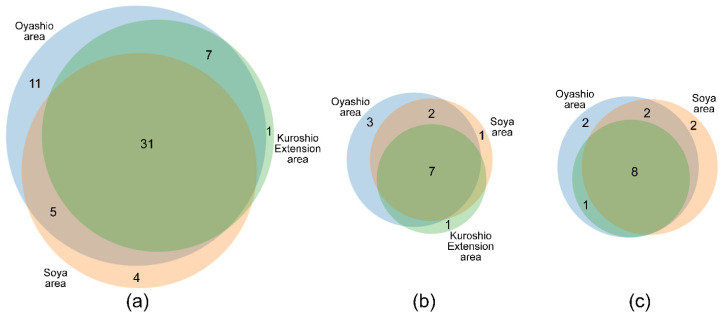
Venn diagram illustrating the overlap in (**a**) total number of species/taxa; (**b**) boreal copepods; and (**c**) subtropical copepods identified in samples from the Oyashio Current (blue), Soya Current (peach), and Kuroshio Extension (light green) areas. Sizes of circles are proportional to the total number of species per area; numerals indicate species richness.

A total of 54 zooplankton species/taxa were identified (Supplementary Table S1). The summer zooplankton mainly comprised amphipods, appendicularians, copepods, chaetognaths, cladocerans, euphausiids, ostracods, salps, and pelagic larvae, with copepods being the most diverse group, accounting for over 50% of the total number of taxa on average. Among small-sized copepods, species of the genera *Paracalanus*, *Pseudocalanus*, and *Oithona* were the most common. Large-sized copepods were represented by *Calanus pacificus*, *Eucalanus bungii*, *Neocalanus cristatus*, *Neocalanus plumchrus*, *Metridia pacifica*, and *Mesocalanus tenuicornis*. The diversity at stations ranged between 16 and 34 species/taxa per station, with the peak of diversity recorded from the warm waters off the southern Kuril Islands. The diversity in the Pacific waters was higher than in the Sea of Okhotsk waters (Supplementary Table S1). The majority of species/taxa (31, or 57%) were found in all three areas (OC, SC, and KE) (Figure 4a). Of the 28 identified copepod species, 16 (or 57%) inhabited subtropical Pacific waters, while the remaining species inhabited subarctic Pacific waters (Figure 4b,c). In terms of faunistic diversity, the summer zooplankton in the OC area was similar to those in the SC and KE areas, while their distribution patterns of abundance and biomass were considerably different.

The total zooplankton abundance varied between 1244.3 and 15,346.2 ind. m^−3^ with a mean value of 5340 ± 876.3 ind. m^−3^. Maximum abundances were recorded from cold waters off the northern Kuril Islands (Supplementary Table S1). The highest copepod abundances were generally found at the stations showing high phytoplankton concentrations; e.g., at stns. 1–4, copepods accounted for more than 98% of the total abundance. Phytoplankton cells were not counted, and phytoplankton concentration was estimated visually in the samples. At the rest of the stations, zooplankton abundances ranged from 3000 to 5000 ind. m^−3^ showing a tendency to increase from south to north. At all stations, small-sized copepods numerically dominated. The contribution of large-sized copepods was higher in the Pacific waters compared to the Sea of Okhotsk area. Other zooplankton taxa rarely accounted for more than 5% of the total abundance.

**Figure 5:**
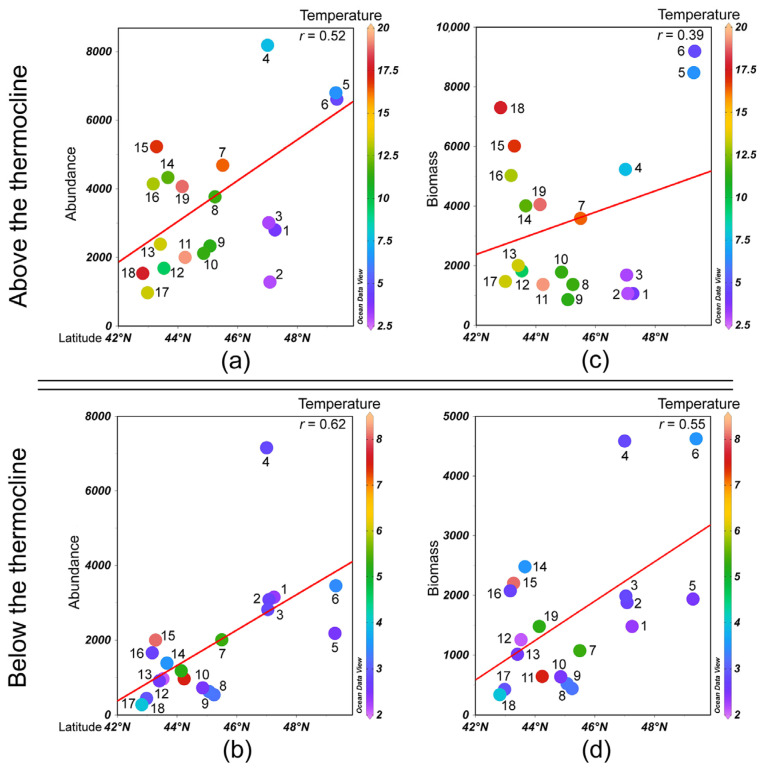
Trends in total zooplankton (**a**,**b**) abundance, ind. m^−3^; (**c**,**d**) biomass, mg m^−3^; (**a**,**c**) above the thermocline; and (**b**,**d**) below the thermocline. The red line indicates linear regression fit. The numerals indicate sampling stations.

The total zooplankton biomass varied between 1383.8 and 14,103.1 mg m^−3^ with a mean of 5158.3 ± 1261.4 mg m^−3^. The highest biomass was recorded from the Pacific waters off the northern Kuril Islands (Onekotan and Simushir Islands) (Supplementary Table S1). In this area, large-sized boreal copepods accounted for more than 80% of the total zooplankton biomass. At the stations off the southern Kuril Island, the zooplankton biomass ranged from 3000 to 6800 mg m^−3^ and was dominated by small-sized copepods. At some stations, other zooplankton taxa contributed significantly to the total biomass: e.g., salps accounted for up to 90% (off Yuri Island), and euphausiids, mainly *Euphausia pacifica* larvae, up to 15% (off Onekotan Island). The total zooplankton biomass, similar to abundance, was higher in the Pacific waters compared to the Sea of Okhotsk waters. It was the highest above the thermocline and decreased with depth, showing a tendency to increase with higher latitude and lower water temperature (Figure 5).

### 3.3. Community Analysis

**Figure 6:**
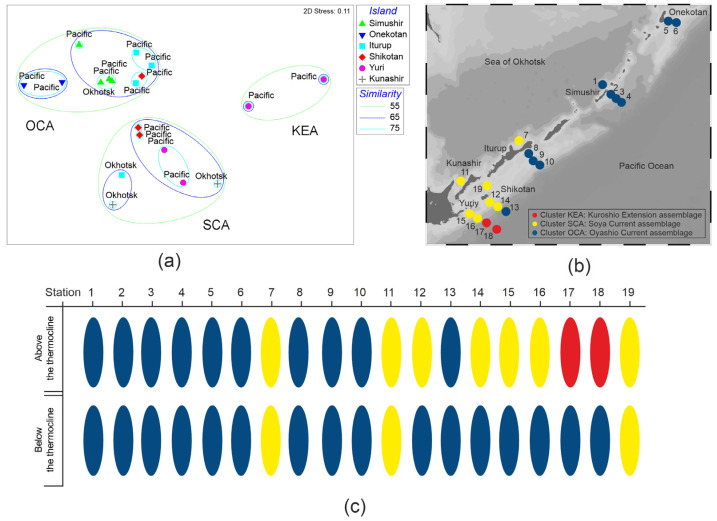
Non-metric multidimensional scaling ordination plot of sampling stations based on (**a**) species abundance; (**b**) spatial distribution; and (**c**) vertical distribution of zooplankton assemblages in the Pacific and Sea of Okhotsk areas along the Kuril Islands identified by Bray–Curtis dissimilarity. Colored circles representing zooplankton assemblages, as identified by nMDS analysis, were superimposed on the station labels.

Three clusters were identified at a 55% similarity by the nMDS ordination of species abundance data (Figure 6a). The stations were combined into clusters, which corresponded to the major currents, i.e., the Oyashio, Soya, and Kuroshio Extension. Accordingly, we referred to them as the Kuroshio Extension assemblage (KEA), the Soya Current assemblage (SCA), and the Oyashio Current assemblage (OCA). The KEA was located in the Pacific waters off Yuri Island; the SCA was distributed in the Sea of Okhotsk waters from Kunashir Island to Iturup Island and from Shikotan Island to Yuri Island in the Pacific waters; the OCA was located in the Pacific waters from north (Onekotan Island) to south (Shikotan Island) (Figure 6b).

The OCA, characterized by small-sized boreal copepods, was observed throughout the water column in the Pacific waters along the Kuril Islands, except for the southern Kuril Islands, where OCA was absent above the thermocline (Figure 6c). The KEA, characterized by salps and copepods, was found above the thermocline in the Pacific waters off the southern Kuril Islands. In the Sea of Okhotsk waters off the southern Kuril Islands, the SCA, characterized by small-sized subtropical copepods, was observed throughout the water column, whereas, in the Pacific waters, it was present only above the thermocline (Figure 6c).

The indicator species/taxa (defined as those with the highest contributions in each cluster) of the KEA were *Metridia pacifica* and salps, which together accounted for 52.7% of the average intra-group similarity (SIMPER; average similarity, 49.6%). The SCA was characterized by the dominance of *Paracalanus parvus* and *Oithona* spp., which together accounted for 79.4% of the average intra-group similarity (SIMPER; average similarity, 55.4%). The OCA was characterized by the dominance of *Pseudocalanus newmani* and *Oithona* spp., which together accounted for 71.6% of the average intra-group similarity (SIMPER; average similarity, 55.6%). The dissimilarity between the tested assemblages was explained by six copepod species and pelagic tunicates, which collectively accounted for 81.2% of the dissimilarity between the KEA and SCA, 80.5% between the KEA and OCA, and 60.6% between the OCA and SCA, as shown by a SIMPER analysis (Table 1). The dissimilarity between the assemblages of the Pacific and Sea of Okhotsk waters was explained by five small-sized copepod species, which collectively accounted for 80% of the dissimilarity, primarily due to the presence of *Paracalanus parvus*, *Pseudocalanus minutus*, and *Oithona* spp., which together accounted for 66% of the dissimilarity (SIMPER; average similarity, 56.2%). The dissimilarity between the assemblages above and below the thermocline was explained by the copepods *Paracalanus parvus*, *Pseudocalanus minutus*, *Pseudocalanus newmani*, *Metridia pacifica*, *Oithona* spp., and salps, which collectively accounted for 83% of the dissimilarity.

### 3.4. Correlation with Environmental Conditions

As our study demonstrated, the zooplankton distribution patterns along the Kuril Islands were depth-stratified, with distinct assemblages occupying layers above and below the thermocline (see Figure 6). The canonical analysis revealed a significant relationship between the biomass of major zooplankton taxa groups and environmental variables (Canonical *R* = 0.73, *p* < 0.001). Strong positive correlations were observed between the biomass of subtropical copepods and both temperature and salinity above the thermocline, indicating an association with the warm waters of the SC and KE (characterized by high temperature and high salinity) (Table 2). In contrast, the biomass of boreal copepods showed strong negative correlations with temperature and salinity above the thermocline, indicating a positive relationship with the OC (characterized by low temperature and low salinity). The biomass of euphausiid larvae and meroplankton (larvae of benthic animals) was positively correlated with chlorophyll *a* concentration and negatively correlated with temperature. The biomass of amphipods and chaetognaths exhibited strong negative correlations with temperature and salinity below the thermocline and moderate positive correlations with water density, indicating their affinity with the OC.

The BIOENV analysis between the environmental variables and the zooplankton abundance showed that significant variations in quantitative distribution could be explained by two environmental variables: temperature and salinity (with the correlation coefficient ranging from 0.54 to 0.59). The other variables did not increase the correlation coefficient (Table 3). These relationships were statistically significant for each of the four models (RELATE; *p* < 0.01). The best combination of variables explaining the variations in abundance was the combination of temperature and salinity below the thermocline.

**Figure 7:**
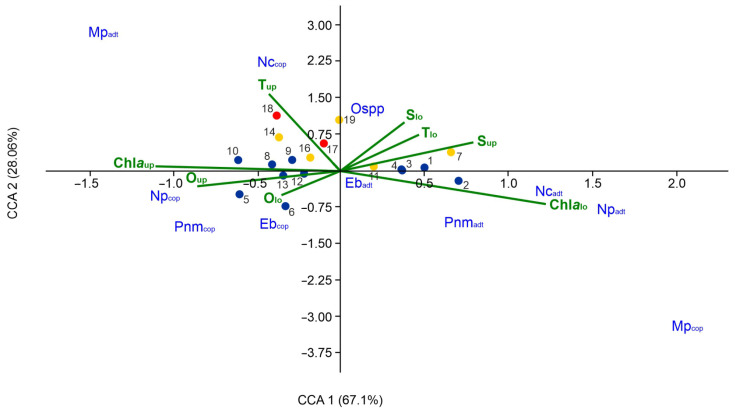
Diagram of the CCA ordination of stations by abundance of boreal copepods in relation to the environmental variables in the study region. Abbreviations of the copepods (in blue) are as follows: Nc, *Neocalanus cristatus*; Np, *Neocalanus plumchrus*; Eb, *Eucalanus bungii*; Pnm, *Pseudocalanus minutus* and *Pseudocalanus newmani*, collectively; Mp, *Metridia pacifica*; Ospp, *Oithona* spp.; adt, adults and stage C5 copepodites, collectively; cop, stage C1–C4 copepodites. Green lines, environmental variables; colored circles, sampling stations. For abbreviations environmental variables, see Section 2.

The CCA method was used to analyze the relationships between the environmental variables and abundance of boreal copepods. The results are presented in Figure 7. The eigenvalues of Axis 1 (CCA1) and Axis 2 (CCA2) were 0.110 and 0.046, respectively, and together these two axes explained 95.1% of the total variance in the species–environmental factor relationship. Only six out of 10 variables (T_up_, T_lo_, S_lo_, Chl*a*_up_, Chl*a*_lo_, and O_lo_) were found to be statistically significant (*p* < 0.05). The species located near the positive side of CCA 1 (67.1%) such as *Oithona* spp. showed a potential relationship with temperature, salinity, and chlorophyll *a* concentration below the thermocline. This suggests that this species is adapted to, or thrives in, habitats with elevated temperatures and salinities and reduced chlorophyll *a* concentrations. Conversely, adults of *Neocalanus cristatus*, *Neocalanus plumchrus*, *Pseudocalanus minutus*, and *Pseudocalanus newmani*, located on the negative side of CCA1, appeared to prefer habitats with low temperatures, low salinities, and high chlorophyll *a* concentrations below the thermocline.

The CCA2 (28.06%) was associated with the factors such as water temperature and chlorophyll *a* and dissolved oxygen concentrations above the thermocline (Figure 7). The distribution of copepodites of *Neocalanus cristatus* towards the upper side of CCA2 suggested that this species is adapted to, or thrives in, habitats with elevated temperatures and may be more sensitive to, or influenced by, oxygen and chlorophyll *a* levels. Copepodites of *Neocalanus plumchrus*, *Pseudocalanus minutus*, *Pseudocalanus newmani*, and *Eucalanus bungii* located on the negative side of CCA2, appeared to prefer habitats with low temperatures and high oxygen and chlorophyll *a* concentrations. In contrast, adults of *Eucalanus bungii*, located near the center of the plot, suggested a tolerance to environmental factors. *Metridia* have a wider range of movement in the water column than other boreal copepods and are more influenced by strong diel vertical migrations than by water temperature and salinity. This may explain the lack of significant correlation between the abundance of *Metridia pacifica* and the environmental factors.

## 4. Discussion

The long-term study of sea current dynamics showed a decreasing trend in the OC water transport rate over recent decades [18,44,45]. The authors assumed that in the coming years, the distribution area of cold OC waters will shrink, while the influence zone of the warm SC branches will expand northward. Our study has provided evidence for this hypothesis: an expansion of the warm subtropical water northward was observed, as the warm and saline SC water from the Sea of Japan extended as far north as Iturup Island, where it had previously been absent [10,16]. At the same time, the extent of the influence zone of the cold OC from the Pacific was reduced. Specifically, the OC water flowing from the northeast either descended below 50 m or deviated southeastward, to the area around Iturup Island. The zooplankton composition in the study region varied markedly depending on the water masses. Small-sized copepods were a dominant component of the warm-water SC and KE distributed in the southern Kuril Islands region, whereas large-sized copepods *Neocalanus* and *Eucalanus*, being lipid-rich prey for fish, were important in the cold-water OC distributed in the northern Kuril Islands region. The high species richness in the Pacific waters off the southern Kuril Islands suggests that the SC and KE assemblages may have formed through the interaction of subarctic and subtropical water masses in this transitional region [14]. Thus, the assemblages may be partially influenced by climate shifts [21,22] and by the increased northward transport of warm water [10,12] resulting in the increased abundance of small-sized species in the northerly areas.

Small-sized cyclopoids *Oithona* are known to be widely and abundantly distributed in the world’s oceans [46,47]. The abundance of *Oithona* species is positively correlated with temperature, i.e., elevated temperatures facilitate their reproduction [48,49]. This suggests that the northward intrusion of subtropical water masses may contribute to an increase in the abundance of cyclopoids that compete with large-sized copepods for phytoplankton. Conversely, the intrusion of subarctic water mass induces an increase in large-sized copepods (such as *Neocalanus plumchrus*, *Neocalanus cristatus*, *Metridia pacifica*, and *Eucalanus bungii*) which are important food items for Pacific salmon [50,51]. *Neocalanus*, being common in the Okhotsk-Kuril region [20,52], formed the bulk of biomass in the cold waters off Onekotan and Simushir islands. All copepodite stages of *Neocalanus* occurred in the waters off the northern Kuril Islands, which indicated the localization of populations of this species in this area. Copepods *Neocalanus* are often associated with diatoms, their food item that provides successful breeding [53,54]. The highest concentrations of phytoplankton (e.g., diatoms *Chaetoceros*) were observed in the waters off the northern Kuril Islands. This indicates that phytoplankton blooms in this area are important for the maintenance of the *Neocalanus* populations. In contrast, in the warm waters off the southern Kuril Islands, these copepods did not form dense patches, their populations did not include the full range of copepodite stages, and they consisted predominantly of adults. This suggests that these older individuals probably originated from *Neocalanus* populations spawning off the northern Kuril Islands and were transported south by the cold Oyashio Current. It is also possible that the southern populations may be only a later succession stage because these processes in warm waters develop usually faster than in cold ones.

In recent decades, the northwestern Pacific Ocean has experienced a shift in the thermal regime toward warming across all regions, depths, and seasons [12]. This trend is primarily driven by variations in heat exchange with the atmosphere and is observed in the interannual dynamics of ice cover, surface water temperature, and geostrophic currents. A weakening of convective water mixing and warming of intermediate water layers has occurred, and, as a result, surface waters have become less productive due to insufficient flux of nutrients from deeper layers [16,18,19,55,56]. During climate change, a rearrangement of pelagic communities has begun: the intensity of spring microalgal blooms decreased in the 2000s and has remained at a low level since then; the total zooplankton biomass has declined sharply, with this trend still continuing [21,23,26,50,57]. In the early 2000s, an increase in the proportion of small-sized copepods was recorded from the Kuril Islands; by 2015, they accounted for up to 30% of the total zooplankton biomass, while the proportion of large-sized copepods showed a declining trend, from 92 to 70% [29]. In the present study, the proportion of small-sized copepods in the southern Kuril Islands area reached 71% of total zooplankton biomass and decreased to 23% further north. The proportion of boreal large-sized copepods off the northern Kuril Islands, and vice versa, reached 66% of total zooplankton biomass and decreased to 20% further south. The maximum biomass of small-sized copepods was observed in warm waters (>13 °C) with relatively low concentrations of chlorophyll *a* (<1 µg L^−1^) and oxygen (<5.5 mL L^−1^) off Iturup and Yuri islands, whereas the maximum biomass of large-sized copepods was in cold waters (<5 °C) with relatively high concentrations of chlorophyll *a* (>1.0 µg L^−1^) and oxygen (>6.5 mL L^−1^) off Onekotan and Simushir islands. The relative proportion of boreal large- and small-sized copepods in the study area was not higher than 41 and 45% of total zooplankton biomass, respectively, indicating that the biomass comprising small-sized copepods remained at a high level, while the biomass comprising large-sized copepods had dramatically decreased compared to that recorded in the early 2000s [27,33,52]. Previously, Miller et al. [53] reported that relatively high-temperature conditions may prevent spawning in females of boreal copepods both in winter and in summer, when C1–C4 copepodites develop, which is the most critical period for their survival [28,58,59]. Most boreal large-sized copepods have biennial life cycles, and spawn in the intermediate layer [60]. Therefore, we hypothesized that the climate shift led to a decrease in the breeding intensity of boreal large-sized copepods in the intermediate layer due to its warming which caused a decline in the abundance of future generations of these copepods and, consequently, a negative trend in the total zooplankton biomass. However, the decrease in the proportion of the biomass of large-sized copepods off the Kuril Islands did not affect the interannual fluctuations in the abundance of juvenile Pacific salmon [29,61]. This contrasts with the findings of previous studies that confirmed the relationship between the average annual abundance of juvenile Pacific salmon and the average abundance of copepods [8]. For example, the abundance of juvenile Chinook salmon (*Oncorhynchus tshawytscha*) and Coho salmon (*Oncorhynchus kisutch*) was negatively correlated with the abundance of small-sized copepods and positively correlated with the abundance of large-sized copepods. It is likely that the changes in pelagic communities will not lead to global shifts in commercial fish stocks, as the abundance of some species can depend on some other factors. For example, the size of the walleye pollock population was determined by intrapopulation regulations (e.g., a negative correlation between the spawning stock and the recruitment to the population), while deep-sea fish such as flounders and halibuts have demonstrated the stability of their stocks under climate change [26]. In addition, it is worth noting that salps were found in the southern Kuril Islands area (7.2 g m^−3^, or 30% in total biomass above the thermocline) and at much higher latitudes than previously assumed [21,30,34,62]. The northward expansion of the salps’ distribution range may cause high food competition for local large-sized copepods, juvenile fish, and euphausiid and hyperiid larvae.

The total zooplankton abundance and biomass above the thermocline were two- or threefold higher than below the thermocline, which is consistent with the previously published data for the Okhotsk–Kuril region [63,64]. Additionally, the total biomass values were twofold higher in the Pacific waters than in the Sea of Okhotsk waters, and the total abundance values did not differ between the Pacific and Sea of Okhotsk waters. The biomass of boreal large-sized copepods was higher in the Pacific waters, while the biomass of boreal small-sized copepods was higher in the Sea of Okhotsk waters. The dominance of boreal large-sized copepods in the Pacific waters may decline in warmer years when they are replaced by their boreal small-sized counterparts. In the vertical distribution, boreal large-sized copepods were concentrated below the thermocline in the Sea of Okhotsk waters, and above the thermocline in the Pacific waters. Thick thermocline, which is usually formed in the Sea of Okhotsk area during the summer season [10,16], may be a key factor responsible for vertical differences between zooplankton assemblages.

## 5. Conclusions

In this study, we have analyzed data on zooplankton habitats, species composition, and spatial and vertical distributions patterns of abundance and biomass obtained during the summer of 2024 with the aim of understanding the ecological relationships between zooplankton and environmental factors. As a result, the spatial and vertical variations in the composition of zooplankton assemblages between the northern and southern Kuril Islands areas have been clearly identified on the basis of the influence of subarctic and subtropical water masses. The water temperature and salinity below the thermocline were the primary environmental factors driving zooplankton distribution patterns. We here show their strong positive or negative correlations with environmental factors, which contribute significantly to total zooplankton biomass, within individual taxa/species that, hence, could be considered indicators of environmental quality. Based on the finding that the occurrence of boreal copepods is negatively correlated with water temperature and salinity, we anticipate a further decrease in the “lipid-rich” biomass in the waters off the southern Kuril Islands due to the predicted sea temperature rise, which is attributed to the strengthening of the KC and the northward expansion of the subtropical zone. Therefore, there is an urgent need for finding a coefficient associated with the proportion of subtropical or boreal species and for the annual integrated monitoring of the highly productive waters to predict the zooplankton response to changes in environmental conditions and control the ecosystem status over different years.

## Figures and Tables

**Table 1:** Results of the SIMPER analysis of zooplankton species/taxa average abundance with contributions (Contr, %) to intra-group similarity (Sim/SD) and between-group dissimilarity (Diss/SD) among the stations for the Oyashio Current assemblage (OCA), Soya Current assemblage (SCA), and Kuroshio Extension assemblage (KEA) off the Kuril Islands. The listed taxa contributed to at least 80% of the dissimilarity between these areas.

Taxa	KEA		SCA		OCA		KEA vs. SCA		KEA vs. OCA		OCA vs. SCA	
	Sim/SD	Contr, %	Sim/SD	Contr, %	Sim/SD	Contr, %	Diss/SD	Contr, %	Diss/SD	Contr, %	Diss/SD	Contr, %
*Oithona* spp.	1.3	17.7	2.1	36.5	2.4	49.6	1.6	22.5	1.9	34.7	1.1	20.5
*Pseudocalanus newmani*					2.1	22.0			0.1	17.0	1.6	11.6
*Pseudocalanus minutus*	0.8	6.7			0.8	9.8	0.9	7.9	0.7	13.1	0.9	14.3
*Paracalanus parvus*	0.6	6.5	2.1	42.2			2.1	33.1			1.8	30.3
*Metridia pacifica*	2.2	26.0	0.8	6.7			1.9	6.3			1.5	5.1
*Eucalanus bungii*									1.7	3.7		
Salpida	0.4	26.6					1.1	14.0	1.0	14.9		

**Table 2:** Spearman’s rank-order correlations between the environmental variables and the biomass (mg m^−3^) of major zooplankton taxa groups. Bold-highlighted values indicate significant (*p* < 0.05) correlations.

Taxa	T_up_	T_lo_	S_up_	S_lo_	Chl*a*_up_	Chl*a*_lo_	O_up_	O_lo_	D_up_	D_lo_
Amphipoda	−0.38	−0.14	−0.23	**−0.52**	0.07	0.16	0.19	−0.08	0.22	0.14
Chaetognatha	−0.28	**−0.62**	−0.41	**−0.52**	0.11	−0.14	0.39	−0.39	**0.55**	**0.53**
Ls-BCop	**−0.60**	−0.22	**−0.55**	−0.51	0.22	0.29	0.44	−0.05	**0.59**	0.23
Ls-SubCop	**0.76**	0.14	**0.61**	−0.06	0.23	**−0.68**	0.03	0.06	**−0.72**	−0.14
Euphausiidae, larvae	**−0.62**	−0.11	−0.01	−0.38	−0.31	**0.78**	−0.01	0.06	**0.59**	−0.01
Salpidae	0.42	0.31	−0.32	−0.33	0.02	0.02	0.41	0.39	**−0.57**	−0.48
Ss-BCop	−0.42	−0.27	**−0.56**	−0.38	0.34	0.28	0.42	0.19	0.44	0.15
Ss-SubCop	**0.76**	**0.47**	**0.53**	0.33	0.11	**−0.47**	−0.29	0.24	**−0.73**	**−0.57**
Meroplankton	−0.43	−0.03	0.12	−0.46	−0.03	**0.59**	−0.29	0.12	**0.48**	0.30

Notes: Ls-BCop, large-sized boreal copepods (e.g., *Metridia pacifica*, *Eucalanus bungii*, *Neocalanus cristatus*, and *Neocalanus plumchrus*); Ls-SubCop, large-sized subtropical copepods (e.g., *Calanus pacificus* and *Mesocalanus tenuicornis*); Ss-BCop, small-sized boreal copepods (e.g., *Pseudocalanus minutus*, *Pseudocalanus newmani*, and *Oithona* spp.); and Ss-SubCop, small-sized subtropical copepods (e.g., *Clausocalanus arcuicornis* and *Paracalanus parvus*). For abbreviations of environmental variables, see Material and Methods.

**Table 3:** Combinations of environmental variables yielding the highest correlations in the BIOENV analysis.

Number of Variables	Correlation Coefficient	Selections
2	0.594	T_lo_, S_lo_
3	0.566	T_lo_, T_up_, S_lo_
4	0.565	T_lo_, T_up_, S_up_, S_lo_
3	0.543	T_lo_, S_up_, S_lo_

## Data Availability

Data are contained within the article and Supplementary Materials.

## Supplementary Materials

The following supporting information can be downloaded at: https://www.mdpi.com/article/10.3390/biology14070827/s1, Table S1: Composition, mean abundance (*N*, ind. m^−3^), and biomass (*B*, mg m^−3^) of zooplankton above and below the thermocline during the research cruise aboard the R/V Akademik Oparin from 17 August to 10 September 2024 along the Kuril Islands.
