# Supplementary material for: Distribution of Summer Zooplankton in the Waters off the Kuril Islands (Northwest Pacific) in Relationship with Environmental Conditions
Source: Biology (Basel). 2025 Jul 8;14(7):827. doi: 10.3390/biology14070827 (PMC12292786; doi:10.3390/biology14070827)
Supplement: Supplementary file 1 [file biology-14-00827-s001.zip › biology-3683917-supplementary.pdf]

Supplementary table S1.

Table S1. Composition, average abundance (*N*, ind. m^{-3}), and biomass (*B*, mg m^{-3}) of zooplankton above and below the thermocline during the research cruise aboard the R/V *Akademik Oparin* from August 17 to September 10, 2024 along the Kuril Islands.

Taxa	Onekotan Island		Simushir Island		Iturup Island		Kunashir Island		Shikotan Island		Yuri Island		Pacific area		Sea of Okhotsk area	
	N	***B***	***N***	***B***	***N***	***B***	***N***	***B***	***N***	***B***	***N***	***B***	***N***	***B***	***N***	***B***
Amphipoda																
Themisto pacifica	2.4/5.1	3.6/7.5	0.6/0.8	0.9/1.2	0.6/2.5	1.1/3.8	0.1/0.1	0.1/0.2	0.4/0.7	0.6/1.1	0.8/0.4	1.1/0.6	0.9/1.8	1.4/2.8	0.1/0.2	0.1/0.4
Hyperia sp.	–	–	–/0.1	–/0.1	–	–	–/0.1	–/0.1	0.1/0.1	0.1/0.1	0.1/–	0.1/–	0.1/0.1	0.1/0.1	–/0.1	–/0.1
Primno macropa	–	–	–	–	0.1/0.1	0.1/0.1	–	–	–/0.1	–/0.1	–	–	0.1/0.2	0.1/0.2	–	–
Monoculodes sp.	–	–	–	–	–/0.2	–/0.7	–	–	–/0.1	–/0.3	–/0.3	–/0.5	–/0.1	–/0.3	–	–
Vibilia sp.	–	–	–	–	–	–	–	–	–/0.1	–/0.1	0.1/0.3	0.1/0.4	0.1/0.1	0.1/0.1	–	–
Paraphronima sp.	–	–	–	–	–	–	–	–	–	–	–/0.2	–/0.3	–/0.1	–	–/0.1	–
Appendicularia																
Oikopleura sp.	–	–	7.2/6.8	2.1/2.1	2.4/0.8	0.6/0.2	88.1/–	132.2/–	0.8/–	1.2/–	14.4/–	21.6/–	5.8/1.2	6.5/0.3	46.4/3.1	66.6/0.7
Chaetognatha																
Parasagitta elegans (<10 mm)	18.2/23.3	27.3/35.1	20.1/20.5	30.1/30.7	2.8/7.3	4.2/11	2.8/4.6	4.2/6.9	16.1/37.8	24.1/56.6	7.9/18.2	11.9/27.3	11.6/20.7	17.4/31.1	9.6/11.3	14.4/17.1
Parasagitta elegans (>10 mm)	2.1/1.7	13.5/11.2	0.6/1.1	4.1/6.5	1.4/2.1	9.1/12.8	0.1/0.5	0.7/3.4	3.7/2.2	24.2/14.3	1.3/1.9	8.4/12.8	1.8/1.8	12.1/12.1	0.2/0.8	1.6/4.8
Cladocera																
Evadne sp.	–	–	–	–	–	–	1.6/–	2.4/–	–	–	0.5/–	0.7/–	0.1/–	0.2/–	0.8/–	1.2/–
Podon sp.	–	–	–	–	–	–	2.2/–	3.3/–	–	–	–	–	–	–	1.1/–	1.6/–
Coelenterata																
Aglantha digitale	8.6/6.8	4.2/3.4	4.3/5.9	2.2/2.9	–/0.6	–/0.3	–/0.1	–/0.1	–/0.2	–/0.1	0.1/0.2	0.1/0.1	2.1/2.3	1.1/1.1	–/2.1	–/1.1

Copepoda																
Acartia longiremis	1.4/3.3	0.9/2.3	4.2/3.5	2.8/2.5	–	–	–	–	3.2/–	3.4/–	–	–	0.8/0.4	0.1/0.3	4.0/3.5	2.8/2.5
Calanus glacialis	20.1/–	60.2/–	–	–	–	–	–	–	–	–	–	–	2.6/–	8.3/–	–	–
Calanus pacificus	–	–	1.2/–	2.7/–	1.7/0.1	4.5/0.4	20.4/1.7	55.1/4.5	15.4/–	41.7/–	6.1/–	16.2/–	5.3/0.1	13.8/0.1	10.8/0.9	27.6/2.5
Calocalanus sp.	–	–	–	–	–/0.1	–/0.1	–	–	–	–	–	–	–/0.1	–/0.1	–	–
Candacia simplex	–	–	–	–	–/0.1	–/0.1	–/0.1	–/0.1	–/0.2	–/0.2	–/0.1	–/0.1	–/0.1	–/0.1	–/0.1	–/0.1
Centropages abdominalis	0.6/–	1.3/–	–	–	–	–	–	–	–	–	–	–	0.1/–	0.2/–	–	–
Clausocalanus arcuicornis	–	–	–	–	–/0.1	–/0.1	40.2/1.6	44.3/1.7	0.6/0.2	0.6/0.2	27.4/6.3	30.1/6.9	7.4/1.4	8.1/1.5	20.0/0.8	22.1/0.9
Chiridius sp.	–	–	–/0.1	–/0.1	0.1/0.1	0.1/0.1	16.4/0.1	20.8/0.1	–	–	–/0.2	–/0.2	0.1/0.1	0.1/0.1	8.0/0.1	10.4/0.1
Eucalanus bungii	304.3/253.3	1368.7/1140	164.7/174.7	738.2/786.3	26.2/16.7	117.1/75.2	0.8/39.8	3.6/179.4	126.9/125.5	571.2/565	46.2/30.7	208.2/138.4	123.5/116.8	556.1/525.8	20.4/46.3	91.9/208.4
Euchaeta sp.	–	–	–	–	–/0.2	–/0.3	0.1/0.2	0.2/0.5	–/0.1	–/0.2	–/0.1	–/0.3	–/0.1	–/0.2	0.1/0.1	0.1/0.2
Euchirella rostrata	–	–	–	–	–/0.1	–/0.1	–	–	–	–	–/0.1	–/0.1	–/0.1	–/0.1	–	–
Labidocera japonica	–	–	–	–	–	–	0.1/0.1	0.2/0.2	–	–	–	–	–	–	0.1/0.1	0.1/0.1
Lucicutia sp.	–	–	–	–	–	–	–/0.2	–/0.5	–	–	–	–	–	–	–/0.1	–/0.2
Mesocalanus tenuicornis	–	–	–	–	4.1/19.1	7.8/36.1	35.3/40.2	53.7/60.4	6.6/1.9	11.3/3.1	140.7/3.4	239.3/5.7	39.9/1.2	68.1/2.1	17.7/38.9	27.1/66.1
Metridia pacifica	3.2/293.3	0.3/29.3	186.2/190.6	18.6/19.1	–/188.1	–/201.6	–/253.3	–/304.1	6.7/349.7	11.1/594.6	133.3/235.5	160.1/282.6	65.5/249.7	47.8/252.7	80.2/217.1	8.1/160.5
Metridia okhotensis	–	–	–/14.5	–/36.5	–/0.1	–/0.5	–	–	–	–	–	–	–/3.5	–/7.6	–/4.7	–/11.9
Neocalanus cristatus	0.4/0.5	2.8/3.7	0.8/4.1	5.6/28.7	0.4/0.6	2.8/4.2	–/0.4	–/3.1	1.7/0.6	13.3/5.1	0.2/0.4	1.8/3.2	0.7/11.1	5.7/8.1	–/2.1	–/14.2

Neocalanus plumchrus	472.3/85.3	2124.5/384.4	178.1/195.4	801.2/879.3	41.6/25.1	187.2/113	12.8/9.3	57.6/41.8	198.6/2.9	894.2/13	59.1/10.5	265.6/47.2	173.2/65.8	779.5/296.3	20.4/47.4	91.9/213.3
Paracalanus parvus	–	–	–	–	800.4/40	880.1/44	1880.2/150.1	2108.0/176.1	261.3/2.6	313.6/3.2	760.7/37.7	912.9/45.3	255.1/8.6	306.1/10.4	1740.4/115.2	1934.1/132.1
Paraeuchaeta elongata	–	–	–/0.1	–/0.3	–/0.5	–/1.1	–	–	0.1/0.2	0.3/0.7	–	–	0.1/0.1	0.1/0.4	–/0.3	–/0.5
Paraeuchaeta sp.	–	–	–	–	0.1/0.2	0.1/0.5	–	–	0.1/0.1	0.1/0.3	–	–	0.1/0.1	0.1/0.2	–/0.2	–/0.4
Pleuromamma robusta	–	–	–	–	–/0.1	–/0.3	–	–	–	–	–/0.3	–/0.8	–/0.1	–/0.2	–	–
Pseudocalanus minutus	3360.1/1074.6	4032.2/1289.6	204.2/230.2	204.3/230.2	81.3/70.6	89.4/77.7	0.2/9.8	0.3/13.4	222.6/190.1	267.2/228.1	462.4/125.2	554.9/150.3	685.4/290.6	810.7/336.2	24.4/62.2	24.4/66.4
Pseudocalanus newmani	344.7/185.3	172.1/92.6	624.6/602.1	312.2/301.1	766.4/82.1	536.2/57.4	160.1/120.3	112.2/84.2	384.2/77.7	307.2/62.2	–	–	469.3/196.4	298.2/104.2	170.1/207.4	102.2/128.8
Oithona spp.	1080.2/466.6	32.4/14.2	2260.4/2428.4	67.8/72.8	1420.1/485.3	42.6/14.5	440.3/426.6	74.1/168.6	1320.1/228.8	105.6/18.3	632.6/236.6	442.8/165.5	1350.1/774.8	166.7/59.6	1000.1/997.6	60.4/107.8
Oncea sp.	6.4/13.3	0.1/0.4	32.5/29.9	0.9/0.8	40.3/17.1	1.2/0.4	16.8/1.3	11.7/0.9	7.4/24.3	0.5/1.9	1.5/0.1	1.1/0.1	21.3/19.6	0.9/0.8	10.4/3.7	5.9/0.5
Sapphirina sp.							1.1/–	0.7/–					–	–	0.5/–	0.3/–
Scolecithricella minor	–	–	–/0.3	–0.1/	–/0.9	–/0.1	–/0.1	–/0.1	–	–	–/0.1	–/0.1	–/0.1	–/0.1	–/1.1	–/0.1
Copepods nauplii	28.2/16	0.2/0.1	67.3/64.8	0.6/0.6	7.1/4.4	0.1/0.1	6.8/1.6	0.1/0.1	3.2/–	0.1/–	10.8/0.2	0.1/0.1	20.1/15.3	0.2/0.1	29.4/24.6	0.2/0.2
Euphausiida																
Euphausia pacifica, egg, nauplii	–	–	–	–	–	–	240.2/0.6	19.2/0.1	–	–	74.1/8.7	0.6/0.1	19.7/1.8	0.1/0.1	120.3/0.3	9.6/0.1
Euphausia pacifica,	352.5/45.3	915.2/117.8	32.1/38.7	51.2/61.9	0.7/0.2	1.1/0.4	0.4/2.4	0.6/3.5	0.3/–	0.4/–	5.4/2	8.2/3.1	56.1/14.6	136.5/29.8	4.2/13.1	6.7/20.8

calyptopis, furcilia																
Euphausia pacifica, juv	9.2/46.8	55.2/280.8	1.6/1.7	10.1/10.4	0.6/0.7	3.9/4.5	0.1/0.5	0.7/2.7	0.7/0.2	4.6/1.7	0.1/0.1	0.3/0.4	1.9/7.4	11.6/44.3	0.3/0.5	2.1/3.1
Bivalvia, larvae	248.2/82.6	4.9/1.6	–	–	1.1/–	0.1/–	2.4/–	0.1/–	–/0.4	–/0.1	–	–	33.1/11.9	0.6/0.2	2.0/–	0.1/–
Cirripedia, cypris	–/3.1	–/0.1	–	–	–/0.6	–/0.1	–/0.2	–/0.1	–/0.2	–/0.1	0.6/0.3	0.1/0.1	0.2/0.7	0.1/0.1	–/0.1	–/0.1
Ctenophora	–	–	–	–	–/0.1	–/0.1	–/0.1	–/0.1	–/0.2	–/0.3	–/0.1	–/0.1	–/0.1	–/0.2	–/0.1	–/0.2
Echinodermata, larvae	168.2/71.3	13.4/5.7	28.4/29.7	0.1/0.1	20.1/0.2	1.6/0.1	60.1/0.4	4.8/0.1	45.3/–	3.6/–	20.3/–	1.6/–	45.4/14.4	3.3/0.8	46.0/15.2	2.6/0.1
Decapoda, larvae	–	–	0.1/–	0.1/–	0.1/–	0.1/–	–	–	–	–	0.4/–	0.3/–	0.1/–	0.1/–	–	–
Gastropoda, larvae	–	–	–	–	–	–	–	–	0.1/0.1	0.4/0.1	0.1/0.1	0.2/0.1	0.1/0.1	0.1/0.1	–	–
Mysida	–	–	–	–	–	–	–	–	–	–	0.2/–	0.1/–	0.1/–	0.1/–	–	–
Ostracoda	–	–	–/0.4	–/0.1	–/3.9	–/3.5	0.4/1.5	0.3/1.1	–/6.4	–/5.1	0.4/5.1	0.3/4.2	0.1/3.7	0.1/3.1	0.2/0.7	0.1/0.6
Pteropoda																
Limacina helicina	280.1/140.2	4.2/2.1	7.2/7.6	7.2/7.6	10.4/1.1	9.3/0.9	2.8/0.6	0.1/0.3	8.2/2.1	0.1/0.1	2.3/0.9	0.1/0.1	44.2/23.1	5.1/2.7	1.4/03	0.1/0.1
Polychaeta, larvae	–/	–/	0.1/0.1	0.1/0.1	0.1/0.1	0.1/0.1	–/0.2	–/0.1	–/1.4	–/0.2	0.1/0.1	0.1/0.1	0.1/0.3	0.1/0.1	0.1/0.2	0.1/0.1
Chromista	–	–	–	–	–/0.2	–/0.1	–/0.1	–/0.1	–	–	–/1.1	–/0.1	–/0.2	–/0.1	–/0.1	–/0.1
Salpida	–	–	–	–	–	–	–	–	0.1/–	0.2/–	390.2/1.1	1950.1/5.3	104.1/0.2	520.1/1.1	–	–
Siphonophorae	–	–	–	–	–/1.2	–/1.5	–/0.2	–/0.4	–/0.7	–/1.4	0.1/0.5	0.1/0.8	0.1/0.6	0.1/0.9	–/0.1	–/0.2
Fish, ova, larvae	–	–	–	–	0.1/0.1	0.1/0.1	6.3/0.1	0.1/0.1	0.2/0.1	0.1/0.1	1.7/0.5	0.1/0.1	0.5/0.1	0.1/0.1	3.1/0.1	0.1/0.1
Total	6707.3/2817.9	8835.4/3421.6	3822.4/4052.5	2262.2/2482.6	3228.6/973.9	1900.4/668.1	3036.8/1069.5	2710.5/1058.8	2634.6/1061.1	2597.5/1580.9	2802.5/729.5	4839.7/902.3	3549.2/18.54.1	3788.1/1738.8	3391.9/1823.8	2518.2/1167.6
Margalef's (*D'*)	3.68		3.95		5.69		5.59		5.80		6.72		5.38		5.19	

Pielou’s evenness (J')	0.83	0.79	0.70	0.73	0.74	0.75	0.76	0.72
Shannon−Wiener (H')	2.55	2.45	2.39	2.45	2.55	2.67	2.54	2.40

Notes: En-dash (–) means that the species was not found. The Pacific area covers sampling stations 2−6, 8−10, and 12−18; the Sea of Okhotsk area covers sampling stations 1, 7, 11, and 19. The numerator – above the thermocline; and the denominator – below the thermocline.
